# The D. D. S. in Europe

**Published:** 1884-05

**Authors:** J. L. Tierney

**Affiliations:** Frankfort-on-the-Main, Germany


### THE D. D. S. IN EUROPE.

BY J. L. TIERNEY, D. D. S., FRANKFORT-ON-THE-MAIN, GERMANY.

A native American graduate on coming to Europe for the first time will be surprised on his arrival to find such a number of so-called American dentists, and he forms the impression that the field is full, when such is not the case. The majority of dentists holding American diplomas are Europeans, and persons of no skill. The public does not and will not recognize them as competent American dentists, and none of the better class will accept their services, but seek out either a native American or a European dentist, who has by his superior skill acquired a first-class reputation. This being the case, one would suppose that an incompetent person holding an American diploma could not harm the dental profession in the eyes of the public. But they do the greatest amount of injury. A few years ago it was a fact recognized by all educated Europeans, that the title of D. D. S. was an evidence of superior ability.

What is the condition to-day? Instead of the D. D. S. being a passport to every social circle, it is day by day becoming more and more marked by suspicion, and this, in my opinion, has been directly brought about by the loose way in which American colleges grant their diplomas. Some of them are either quite reckless as to whom they confer degrees upon, or are altogether too credulous for men in their position. I can point out many instances of European graduates of American Dental Colleges who know scarce a word of English. How could such men understandingly attend lectures? Others have told me they were never able to fill a tooth with gold while in the college, and are not, even at this day, able to do the most ordinary operations, except in plastics. Conferring degrees upon such persons has naturally brought the honest, competent graduates into disrespect.

A few years ago a dentist of this city, now deceased, who was recognized in all Europe as a man of superior ability, a graduate of one of our best schools, had in his employ a male servant, who never did any work in the laboratory or operating room, except to clean the rooms after office hours. One day he notified his employer that he would in a few days marry another servant in the house. This was not agreeable to the doctor. He dismissed both of them, and they were married immediately. A few days after this occurrence the doctor learned that his former door-servant had gone to America to study dentistry. This was rather a surprise to him, but greater was his astonishment to learn, barely six months after, that his servant had returned from America with a diploma (from the same college at which the doctor himself had graduated), and had commenced practice in the same city with his former employer; and what was more, at his matriculation he had named as his preceptor his former master, the well-known dentist, and had represented that he had been in his employ several years. This was quite true, but it was simply as a servant. Had the dean required him to produce a certificate from his preceptor, certifying that what he represented was true, he could not have matriculated as a five-year practitioner.

I will cite another recent case in this city. A young journeyman barber, while shaving one of my patients, remarked that he was about to leave his employer. Imagine my patient's surprise on being informed only six months after, that this same barber had just returned from America with a genuine dental diploma from one of our reputable colleges. Being a medical man, he inquired of me how such a person could receive a diploma. I told him I was unable to explain such conduct on the part of such a highly respectable dental college. These, and many other cases, force me to protest in the name of all respectable graduates against this slipshod system of allowing Europeans to graduate without passing the same examination as Americans. If they are so unfortunate as not to be able to understand the lectures, from want of knowledge of the English language, they ought to be obliged to remain longer at college; say three, four, or even five years, or until they become qualified for their degree. Readers of American dental journals are sick and tired of hearing of the efforts of dental societies to erect a higher standard of teaching, when these same societies make no attempt to check the indiscriminate granting of diplomas, regardless of qualification. The system of teaching is quite satisfactory. What is required is more strict examinations, and the placing of all students upon the same level. Europeans should be obliged to pass the same examination as do native Americans. It is not the Buchanan or Morrison bogus diploma factories that are doing the most injury. There is an opportunity for knowing their status, and for openly denouncing them. But it is the reckless manner in which supposed-to-be reputable colleges are conferring degrees upon Europeans, that is demoralizing American dentistry abroad. Colleges seem to act upon the principle that, as the foreign graduates are to live out of the country, they will not probably be called to an account for conferring upon them unearned diplomas.

These are plain statements, but they are true. This protest should have come from other than an individual source, but as it has not been made, I myself enter it, and when called upon to produce the evidence to substantiate my charges, it shall be forthcoming.

